# *APOE* gene polymorphism in long-lived individuals from a central China population

**DOI:** 10.1038/s41598-017-03227-5

**Published:** 2017-06-12

**Authors:** Guodong Liu, Xiang Liu, Pulin Yu, Qi Wang, Hua Wang, Chenfang Li, Guangming Ye, Xiaoling Wu, Chunling Tan

**Affiliations:** 1 Department of Geriatrics, Zhongnan Hospital, Wuhan University, Wuhan, China; 2 Department of Stomatology, the First Affiliated Hospital, Hainan Medical University, Haikou, China; 3 National Institute of Geriatric Medicine, Beijing Hospital, Beijing, China

## Abstract

Previous studies from European and East Asian cohorts reported conflicting results over whether and how the frequencies of the three common alleles, *ε*2, *ε*3 and *ε*4, of the apolioprotein E gene *(APOE)*, in long-lived individuals differ from those in younger age groups. This study was the first to analyse these frequencies of long-lived individuals from central China. Genotyping of *APOE* alleles and genotypes was carried out in 70 long-lived individuals and 204 younger controls. No difference in the frequency of any *APOE* allele or genotype was found between the long-lived participants and their younger controls, but the long-lived group seemed to have a higher *ε*4 frequency (15.71%) than the 24–50 and 51–75 age groups (10.2% and 11.32%, P > 0.05). Notably, when compared with two other Chinese studies, the central China long-lived group had a higher *ε*4 frequency than its southern and eastern China counterparts (15.71% *vs*. 2.82% and 2.54%, P < 0.05). It is not clear to what extent population substructure or lifestyles contributed to these divergent findings. A clear understanding of the contribution of *APOE* polymorphisms to longevity in the Han Chinese population may be achieved only through large scale studies with participants from well-defined regional clusters.

## Introduction

Population ageing is accelerating in both developed and developing countries. As a result, the number of long-lived individuals, including nonagenarians and centenarians, has been rising at an unprecedented pace. Favorable environmental factors, improved medical care and genetic influence are generally considered the major determinants of longevity^1^. The estimated contribution of genetics to life span is between 20–30%^2^. Of many candidate longevity genes that have been investigated, the apolioprotein E gene *(APOE)* is among the very few that have been consistently identified to be associated with human longevity^3–5^. Protein products encoded by *APOE* play important roles in lipid metabolism and neuronal maintenance and repair, and are involved in the development of several cardiovascular and neurodegenerative disorders^6^. The *APOE* gene has three common alleles, known as *ε*2, *ε*3 and *ε*4, which generate three homozygous (*ε*2/*ε*2, *ε*3/*ε*3 and *ε*4/*ε*4) and three heterozygous (*ε*2/*ε*3, *ε*2/*ε*4 and *ε*3/*ε*4) genotypes^7, 8^.

The *ε*3 allele occurs with the highest frequency and all three alleles show various degrees of variability in frequency among world populations. Based on the relationship between food sources and *APOE* allele distribution patterns, researchers have speculated that, evolutionarily, *ε*4 belongs to the ancestral allele, *ε*3 serves as the transitional allele, and *ε*2 represents the selectively favored allele^9^. Accordingly, long-lived individuals would be expected to have higher frequencies of *ε*2 and lower frequencies of *ε*4 than those in younger age groups. Two genome-wide association studies (GWAS), one on German and the other on Dutch long-lived individuals, mostly nonagenarians, identified the *APOE* allele *ε*4 as a major genetic determinant of survival into old age^4, 10^. A subsequent large-scale linkage analysis with nonagenarian sibling pairs from eleven European countries also showed that genetic variation at the *APOE* gene locus was significantly associated with longevity^11^. The influence of genetics is thought to become more prominent as people reach exceptional longevity, i.e., beyond age 100^12, 13^. However, a large number of studies involving centenarians with ethnically matched younger controls in several European and East Asian cohorts have produced conflicting results. Some reported a positive association of *ε*2 and a negative association of *ε*4 with exceptional longevity^14, 15^, whereas others showed negative findings^16, 17^. The reasons for such discrepancies have yet to be fully explained. Sampling errors, sample types and population variation may be complicating factors. It is also possible that the relatively moderate contribution of genetics to longevity makes the detection of responsible genes difficult.

Studies on *APOE* allele frequencies in long-lived individuals in the Chinese population are very limited. A study with participants from eastern China found a decreased frequency of *ε*4 but no increased frequency of *ε*2 in people aged 85 and beyond, compared with younger age groups^18^. In another study with a population in a southern province, a higher frequency of *ε*3 and a lower frequency of *ε*4 were observed in long-lived individuals^19^. Since the Chinese population shows considerable genetic diversity across different geographical regions^20^, far more research is needed to obtain data that may shed light on the characteristics of the *APOE* gene polymorphism and its significance in longevity. In this study, we analysed *APOE* allele and genotype frequencies of long-lived individuals from a central China population and compared our findings with results from other studies.

## Results

### General information and health status of long-lived participants

There were 14 men and 56 women in the long-lived individuals, aged 90–104, with an average age of 99.12 ± 3.2, and 93 men and 111 women in the younger healthy controls, aged 24–75, with an average age of 56.5 ± 9.3. Most of the long-lived participants had one or more major diseases, as shown in Table 1. The diagnoses were based on medical history, physical examinations and laboratory tests. The diet of the subjects consisted primarily of rice and vegetables, with very limited amounts of meat and eggs. More than half of them had a daily intake of calories below 1200 and showed signs of various degrees of malnutrition. About 70% of the subjects suffered sarcopenia, especially in the lower extremities, and had difficulty standing or walking unaided.

**Table 1:** Major Diseases in Long-Lived Participants.

Disease	n	%
Hypertension	57	81.43
Hyperlipidemia	27	38.57
Osteoarthritis	15	21.43
Heart disease	14	20.00
Stroke	3	4.29

### APOE allele and genotype frequencies in long-lived participants and controls

Allele and genotype frequencies of *APOE* in long-lived individuals and their younger controls are shown in Table 2. The distribution of *APOE* genotypes was consistent with Hardy-Weinberg equilibrium (P > 0.05 for each group). The most frequent allele was *ε*3 (78.57%, 83.16% and 82.55% for the long-lived group, the 24–50 age group and the 51–75 age group, respectively), and the most frequent genotype was *ε*3/*ε*3 (60.00%, 67.35% and 66.98% for the long-lived group, the 24–50 age group and the 51–75 age group, respectively). The frequency of *ε*4 in the long-lived group seemed higher than the frequencies in the 24–50 and the 51–75 age groups (15.71% vs. 10.2% and 11.32%), but there was no statistically significant difference between them (P > 0.05), nor was there any statistically significant difference between the long-lived group and either of the control groups in the frequency of any of the other alleles or genotypes (P > 0.05).

**Table 2:** Frequencies of *APOE* Alleles and Genotypes in Long-Lived Individuals and Controls.

	Long-Lived n (%)	Control (ages 24–50) n (%)	Control (ages 51–75) n (%)	p
Allele				
*ε*2	8 (5.71)	13 (6.63)	13 (6.13)	0.941
*ε*3	110 (78.57)	163 (83.16)	175 (82.55)	0.522
*ε*4	22 (15.71)	20 (10.2)	24 (11.32)	0.285
Genotype				
*ε*2/*ε*2	0 (0.00)	0 (0.00)	0 (0.00)	—
*ε*2/*ε*3	7 (10.00)	12 (12.24)	12 (11.32)	0.903
*ε*3/*ε*3	42 (60.00)	66 (67.35)	71 (66.98)	0.554
*ε*4/*ε*2	1 (1.43)	1 (1.02)	1 (0.94)	0.954^
*ε*4/*ε*3	19 (27.14)	19 (19.39)	21 (19.81)	0.415
*ε*4/*ε*4	1 (1.43)	0 (0.00)	1 (0.94)	0.394^

^^^Fisher’s exact test was used; Pearson’s *χ* ^2^ test was used for the rest.

### Allele/genotype frequencies in long-lived individuals from three regions of China

Since the two studies about other regions of China had reported differences in allele and genotype frequencies of *APOE* between long-lived individuals and their younger controls, we decided to compare the long-lived groups of the three studies (including ours) in one analysis and the three control groups in another. Central China was represented by participants from Zhongxiang, Hubei Province. Data for southern China came from a study with participants from the rural Yongfu County of Guangxi Province. The long-lived group was composed of 93 male and 226 female subjects, aged from 90 to 108 years. The Eastern China study contained participants from Shanghai and a mostly urban area of Jiangsu Province. The very old group had 59 subjects, 5 male and 54 female, aged from 85 to 108 years.

The distribution of allele and genotype frequencies of *APOE* in long-lived individuals from three different regions of China can be seen in Table 3. For most of the alleles and genotypes, Pearson’s *χ* ^2^ test was used for comparison; for genotypes where the theoretical frequency was very small, Fisher’s exact test was used. The central China group showed higher frequencies of *ε*4 (15.71% *vs*. 2.82% and 2.54%) and *ε*4/*ε*3 (27.14% *vs*. 4.39% and 5.08%), but lower frequencies of *ε*3 (78.57% *vs*. 90.44% and 88.98%) and *ε*3/*ε*3 (60.00% *vs*. 82.45% and 77.97%), than both the southern China group and the eastern China group (P < 0.05 for each). No difference was found between the southern China group and the eastern China group.

**Table 3:** Distribution of Allele/Genotype Frequencies in Long-Lived Individuals from Central, Southern and Eastern China.

	Central China n (%)	Southern China n (%)	Eastern China n (%)	p
Allele				
*ε*2	8 (5.71)	43 (6.74)	10 (8.47)	0.675
*ε*3	110 (78.57)	577 (90.44)*	105 (88.98)*	0.00037
*ε*4	22 (15.71)	18 (2.82)*	3 (2.54)*	0.00000000039868
Genotype				
*ε*2/*ε*2	0 (0.00)	2 (0.63)	0 (0.00)	1.000^
*ε*2/*ε*3	7 (10.00)	37 (11.60)	10 (16.95)	0.433
*ε*3/*ε*3	42 (60.00)	263 (82.45)*	46 (77.97)*	0.000198
*ε*4/*ε*2	1 (1.43)	2 (0.63)	0 (0.00)	0.64^
*ε*4/*ε*3	19 (27.14)	14 (4.39)*	3 (5.08)*	0.0000000012388
*ε*4/*ε*4	1 (1.43)	1 (0.31)	0 (0.00)	0.493^

*Statistically different from the central China group. ^^^Fisher’s exact test was used; Pearson’s *χ* ^2^ test was used for the rest.

### Allele/genotype frequencies in normal controls from three regions of China

Allele and genotype frequencies in younger controls from the three regions were also compared. Controls from the southern China study had 506 subjects aged between 40 and 79, with an average age of 52.98, and 272 were male and 234 were female. Controls from the eastern China study included 1503 individuals, of whom 1262 were female and 241 were male, with ages ranging from 20 to 84 and an average age of 65.4. Table 4 shows the distribution of allele and genotype frequencies of *APOE* in younger controls from the three regions. Comparison of frequencies for all the alleles and genotypes was conducted with Pearson’s *χ* ^2^ test. The central China group had higher frequencies of *ε*4 and *ε*4/*ε*3 (for *ε*4, 10.78% *vs*. 7.68%; for *ε*4/*ε*3, 19.61% *vs*. 12.04%), and a lower frequency of *ε*3/*ε*3 (67.16% *vs*. 73.79%) than the eastern China group (P < 0.05 for each). Meanwhile, the southern China group had higher frequencies of *ε*4 (11.07% vs. 7.68%) and *ε*4/*ε*4 (2.77% vs. 0.86%), but lower frequencies of *ε*3 (79.35% vs. 85.36%) and *ε*3/*ε*3 (64.82% vs. 73.79%), than the eastern China group (P < 0.05 for each). There was no difference in any allele or genotype between the central China group and the southern China group.

**Table 4:** Distribution of Allele/Genotype Frequencies in Younger Controls from Central, Southern and Eastern China.

	Central China n (%)	Southern China n (%)	Eastern China n (%)	p
Allele				
*ε*2	26 (6.37)	97 (9.58)	209 (6.95)	0.015
*ε*3	338 (82.84)	803 (79.35)†	2566 (85.36)	0.000037
*ε*4	44 (10.78)	112 (11.07)†	231 (7.68) *	0.001
Genotype				
*ε*2/*ε*2	0 (0.00)	4 (0.79)	9 (0.60)	0.457
*ε*2/*ε*3	24 (11.76)	76 (15.02)	167 (11.11)	0.065
*ε*3/*ε*3	137 (67.16)	328 (64.82)^†^	1109 (73.79)*	0.000257
*ε*4/*ε*2	2 (0.98)	13 (2.57)	24 (1.60)	0.239
*ε*4/*ε*3	40 (19.61)	71 (14.03)	181 (12.04)*	0.009
*ε*4/*ε*4	1 (0.49)	14 (2.77)†	13 (0.86)	*0.002*

Statistically different from the central China group. ^†^Statistically different from the eastern China group.

## Discussion

A major finding of the current study is that there was no difference in the frequency of any *APOE* allele or genotype between the long-lived participants and the younger controls from central China. Our results are in contrast to findings from two other studies involving long-lived Han Chinese individuals. The two studies, one with participants from southern China and the other with participants from eastern China, both reported decreased *ε*4 in long-lived individuals but differed in their findings with the frequencies of *ε*2 and *ε*3^18, 19^. Studies from other countries concerning the polymorphism of *APOE* in long-lived individuals have had mostly centenarians as their subjects and some of them have also observed a lower frequency of *ε*4 with or without a concurrent increase in the frequency of *ε*2 in European and Japanese cohorts^14, 15^. Since it is suggested that genetic components contribute more to the life span of longer-lived individuals, it is not clear whether or how centenarians differ from nonagenarians in the frequency of *APOE* alleles and genotypes. Although evidence in support of certain *APOE* alleles as influence factors for longevity seems strong, the issue is far from settled. At least two reasons can account for why some studies, including the present one, have failed to uncover *APOE* alleles or genotypes in long-lived individuals that show either higher or lower frequencies than those individuals in younger age groups. First, although it is possible that *APOE* serves as a major contributing factor for an extended life span in some people, longevity may be the outcome of cumulative effects of multiple genes in many other cases. Second, the rapid increase in the numbers of centenarians in the past several decades around the globe clearly indicates that many non-genetic factors are more important determinants of longevity, such as healthcare, psychological traits and life style^21^.

Another notable finding is that the long-lived individuals in Zhongxiang, who came from a Han Chinese population in central China, had higher frequencies of *ε*4 than their counterparts from southern and eastern China. Also, compared with younger controls from the same region, they had higher frequencies of *ε*4 (15.71% vs. 10.78% for younger controls; 15.71% vs. 11.32% for older controls), although the differences did not reach statistical significance. Despite contrary results from studies on other populations, this finding suggests that *ε*4 is at least not a negative trait for this group. It is possible that this trait is somehow associated with their longevity. Our assessment of other aspects of their lives revealed hardly any favorable factors. As rural residents with limited financial means, most of them rarely sought medical services at clinics or hospitals. Their living conditions, including housing facilities, homecare services and amenities, were generally poor. Given that their daily calorie intake was unusually low, the interaction between long-term dietary restriction, longevity and *ε*4 is worth further examination. The long-lived individuals in our study also had lower frequencies of *ε*3 and *ε*3/*ε*3 and higher frequencies of *ε*4/*ε*3 than those from southern and eastern China. Additionally, control groups from the three regions showed differences in certain allele and genotype frequencies, such as *ε*3/*ε*3. Since these studies are the only three from the Han Chinese population concerning allele and genotype frequency distribution of *APOE* in long-lived individuals, far more information is needed before any conclusion can be made of how much genetic substructure contributed to the divergent findings. Although genetic variation within the Han ethnicity is not as great as that among individuals of European descent^22^, there is evidence that its allele frequency distribution displays clear geographic patterns. Two GWAS have shown that the genetic differentiation among the Han Chinese population follows a strong north-south trend^23, 24^. The genetic substructure can also be subdivided into three clusters, the northern Han, the central Han, and the southern Han^24^. However, the substructure of large urban centres exhibits enormous diversity and therefore cannot be defined by their geographic locations, presumably as a result of heterogeneous population origins^23^. Therefore, results from the eastern China study, with most participants from urban areas, may not best represent characteristics of the *APOE* polymorphism of that region.

Apart from the association of *APOE* with longevity revealed by genetic studies, the alleles of this gene have been demonstrated to produce different and sometimes opposite effects in lipid metabolism. A primary function of *APOE* products is their involvement in the transport and clearance of lipids, such as low-density lipoprotein cholesterol, high-density lipoprotein cholesterol, very low-density lipoprotein cholesterol and triglycerides^6^. The *ε*4 allele contributes to elevated total cholesterol and low-density lipoprotein cholesterol, whereas *ε*2 is closely correlated with low levels of triglycerides and low-density lipoprotein cholsterol^25, 26^. The *ε*4 allele shows enhanced responsiveness to dietary fats and promotes cholesterol absorption^27^. Accordingly, individuals carrying *ε*4 are at increased risk for coronary heart disease and Alzheimer’s disease^26, 28^. However, other studies have found that the frequency of *ε*4 varies with latitude^29, 30^. One study, in particular, demonstrated higher *ε*4 frequencies near the equator and in the far north, which represent hot and cold environments, respectively. In addition, the geographical pattern of the *ε*4 frequency was associated more with latitude than with population substructure^31^. These observations led the authors to suggest that, with elevated metabolic rates, demand for cholesterol might drive selection for higher circulating cholesterol concentrations and thus the *ε*4 allele^31^. Among Europeans, *ε*3 and *ε*4 are negatively correlated and exhibit a south-north shift, with higher *ε*3 and lower *ε*4 in southern Europe and lower *ε*3 and higher *ε*4 in northern Europe. In contrast, *ε*2 does not show a clear pattern, with roughly even distribution across the region^9^. In Asian countries, the frequencies of *ε*3 and *ε*4 are comparable to those in European countries, with the latter showing wider regional variation. The frequencies of *ε*2 appear to form a gradient descending from south to north^9^. For the Chinese population, the frequency of *ε*2 reported from previous studies ranged from about 7.5% to slightly over 10%^32, 33^. In our study, the frequencies of *ε*2 in the long-lived group and the control group were 5.71% and 6.37%, respectively, similar to those from the eastern China study. The southern China study had a higher frequency in the control group, which is consistent with the south-north gradient.

It needs to be pointed out that there are obvious limitations associated with this study. One of our selection criteria required participants to possess a certain level of cognitive capability so that they would be able to cooperate during interviews and physical examinations, but selection bias could have been introduced because of it. Also, compared with most studies of its kind, this was a small convenient sample. The reason we chose to study rural residents was that they are highly representative of the regional genetic substructure since the degree of population mixing is largely negligible. Despite vast improvement in life expectancy over the past decades, as a developing country with a majority rural population, China still has a considerably lower density of people reaching exceptional longevity than economically more developed countries. Consequently, it remains a challenge to recruit large numbers of long-lived individuals in ageing research. The discrepancies in allele and genotype frequencies of *APOE* in participants from the three studies could have been in part due to relatively small sample sizes, but alternative explanations, both genetic and non-genetic, cannot be rule out, considering that very limited data on this subject have been acquired from the Han Chinese population and that conflicting findings from other populations have yet to be resolved. Our findings on the distribution of *ε*4 and other allele and genotype frequencies in long-lived individuals in central China may reflect the aggregate effects of genetic substructure and lifestyle factors of this region. To achieve a clear understanding of regional and individual differences in frequency distribution of *APOE* alleles and genotypes and the contribution of *APOE* polymorphisms to longevity in the Han Chinese population, concerted efforts should be made to conduct studies on larger scales with participants from well-defined regional clusters to validate the findings of this study and the other two studies involving long-lived individuals from southern and eastern China.

## Methods

### Participants

The study was conducted during the period from January 2013 to July 2016, enrolling a total of 70 long-lived individuals, including 20 nonagenarians and 50 centenarians. The participants were residents of Zhongxiang County, Hubei Province, in central China. Zhongxiang is located in central Hubei, with a largely rural population. In order to provide government assistance to senior residents of advanced age, the Zhongxiang Bureau of Civil Affairs established a database for long-lived individuals (aged 90 and over) of the county about 30 years ago and makes regular updates as new information becomes available. To be eligible for admission into the study, potential participants first went through age verification, which required a resident identification card bearing the date of birth and personal information in the county bureau’s database. Since this study was part of a project that was intended to assess the health status and quality of life of long-lived individuals in Zhongxiang and the neighboring rural areas and required participants to be able to communicate well, only individuals without evident cognitive impairment were included in this study. For younger controls, 204 individuals aged between 24 and 75 years were recruited. The controls were local residents who had undergone regular health screening showing no major diseases and had no knowledge of family members who had lived beyond age 90. The controls were divided into two groups, one with ages between 24 and 50 and the other with ages above 50. Their frequencies of the *APOE* alleles and genotypes were compared with those of the long-lived individuals. Before enrollment, informed consent was obtained from all participants of the study. The research protocol was approved by the ethics committee of Zhongnan Hospital and was in accordance with the guidelines of the Declaration of Helsinki (2008).

### Health assessment

Health assessment for participating long-lived individuals was conducted at their homes and included interviews and physical examinations. Interviews were composed of questionnaires and follow-up questions about participants’ medical and family history, cognitive function and activities of daily living. Physical examinations collected data on body weight, height, blood pressure, heart rate, and musculoskeletal function. Information about quality of life and nutritional status was also gathered. Venous blood samples were drawn into blood collection tubes (with EDTA as the coagulant) from participating long-lived individuals before breakfast and placed in a container filled with dry ice. After arriving at the laboratory, samples were stored in a freezer at −80 °C until further use. In addition to genotyping, the samples were also assayed to establish blood lipid profiles.

### Genotyping

Genomic DNA was extracted from collected venous blood samples using a commercial kit (QIAamp DNA Blood Mini Kit, Qiagen, Shanghai, China), which provided a silica membrane-based DNA purification method. The assays were performed with strict adherence to the protocols and instructions specified by the manufacturer. APOE genotyping was conducted using a detection kit (GeneChip Assay, Sinochip, Zhuhai, China) to detect the 112 (rs429358) and 158 (rs7412) positions. First, all samples were amplified with the Verti^tm^ DX Thermal Cycler (Life Technologies, Singapore) (45 cycles, 94 °C for 30 seconds and 65 °C for 45 seconds); then the amplified products were assayed by the fully automated GeneChip detection system (Sinochip, Zhuhai, China). All genotyping results were obtained from the GeneChip automated analysis system.

### Comparison with data from studies about other regions of China

In order to see precisely how our findings would contrast with those from studies involving populations from other regions of China, we selected two studies and analysed our results against theirs on *APOE* allele and genotype frequencies. In fact, the two Chinese studies were the only ones that had dealt with the APOE polymorphism in long-lived Han Chinese people, one with subjects from eastern China^18^ and the other with subjects from southern China^19^. Since controls from these studies did not have exactly the same age range as ours, we treat controls from each study as a single group and compared their distribution of *APOE* allele and genotype frequencies.

### Data analysis

SPSS version 23.0 for Windows (SPSS, Inc., Chicago, USA) was used for all statistical analyses and the significance level was set at 0.05 for all tests. The *χ* ^2^ test was conducted for Hardy-Weinberg equilibrium. For comparison of *APOE* allele or genotype frequencies between long-lived individuals and their younger controls and comparison of data from different regions, either Pearson’s *χ* ^2^ test or Fisher’s exact test was used, depending on the theoretical frequency.
